# Association between Internet Use for Health Information and Self-reported Alzheimer’s Disease Diagnosis among Older Adults in the United States: Racial and Gender Disparities

**DOI:** 10.21203/rs.3.rs-10570409/v1

**Published:** 2026-08-27

**Authors:** Henry M. Zeidan, Gulzar H. Shah, Iman H. Zeidan, Hani M. Samawi, Jeffery A. Jones

**Affiliations:** Jiann-Ping Hsu College of Public Health, Georgia Southern University; Jiann-Ping Hsu College of Public Health, Georgia Southern University; McLaren Health Care, Central Michigan University; Jiann-Ping Hsu College of Public Health, Georgia Southern University; Jiann-Ping Hsu College of Public Health, Georgia Southern University

**Keywords:** Alzheimer’s disease, digital health, internet use, health equity, aging, racial and ethnic disparities, socioeconomic status, public health

## Abstract

**Background:**

Alzheimer’s disease (AD) disproportionately affects racial and ethnic minority populations, reflecting broader inequities in access to diagnosis, treatment, and long-term care. As digital health technologies become increasingly widespread, understanding whether internet use for health information is associated with Alzheimer’s disease diagnosis may inform strategies to address disparities in aging populations.

**Aim:**

This study examined the association between internet use for health or medical information and AD diagnosis among older adults in the United States and assessed whether this association varied by race/ethnicity, gender, and socioeconomic status.

**Subject and Methods:**

Data were drawn from Round 13 (2024) of the National Health and Aging Trends Study, a nationally representative survey of adults aged 65 years and older. Internet use for health information was assessed via self-report, binary and multivariable logistic regression models estimated adjusted odds ratios for AD diagnosis, controlling for sociodemographic characteristics.

**Results:**

Internet use for health information was significantly associated with Alzheimer’s disease diagnosis. Stratified analyses by race/ethnicity and gender showed consistent patterns, although no statistically significant interaction effects were observed.

**Conclusion:**

These findings suggest that digital health engagement is associated with Alzheimer’s disease diagnosis among older adults and underscore the importance of equitable and culturally responsive access to digital health resources to address persistent disparities in cognitive aging.

## 1. Introduction

Population aging, the growing burden of chronic disease, and rapid digitalization of healthcare are converging to create major public health challenges. Alzheimer’s disease (AD) is among the most consequential of these challenges. In the United States, approximately 6.7 million adults aged 65 years and older are currently living with AD, a figure projected to nearly triple by 2050 [1]. AD imposes substantial human and societal costs, with annual expenditures exceeding USD 345 billion for healthcare services and unpaid caregiving [1, 2].

At the same time, access to health information and services has increasingly shifted to digital platforms. Internet-based health information seeking is now widespread, with roughly 80% of U.S. internet users reporting that they have searched online for health or medical information [3, 4]. For individuals managing chronic conditions, digital health resources may support self-management, improve health literacy, and enhance engagement with healthcare systems [5, 6]. However, these potential benefits are not equally distributed.

Persistent disparities in internet access and digital literacy—commonly referred to as the digital divide—remain strongly patterned by age, race and ethnicity, education, and socioeconomic status [7]. Among older adults, Black and Hispanic individuals are substantially less likely to use the internet for health information than their White counterparts [8]. These disparities reflect broader structural inequities, including unequal access to broadband infrastructure, cumulative educational disadvantage, and reduced trust in healthcare systems [8, 9]. Recent research has expanded understanding of digital health engagement among older adults beyond simple measures of internet access. Digital health engagement encompasses a range of activities including seeking health information online, using patient portals, communicating electronically with healthcare providers, participating in telehealth services, and utilizing mobile health applications. Studies conducted during and after the COVID-19 pandemic demonstrated a substantial increase in digital health utilization among older adults, particularly for healthcare communication and information seeking. However, significant disparities persist according to age, race and ethnicity, educational attainment, income, and geographic location [10–12]. Digital literacy and eHealth literacy have emerged as critical determinants of successful engagement with digital health resources. eHealth literacy refers to an individual’s ability to locate, understand, evaluate, and apply health information obtained through electronic sources. Older adults with higher levels of eHealth literacy are more likely to engage in preventive health behaviors, participate in shared decision-making, and effectively manage chronic diseases [13, 14]. Conversely, limited digital literacy may exacerbate existing health disparities by restricting access to timely health information and healthcare services.

Theoretical perspectives from cognitive reserve theory and technology adoption frameworks provide a basis for understanding the potential relationship between internet use and cognitive health. Cognitive reserve theory proposes that mentally stimulating activities strengthen neural networks and increase resilience to age-related cognitive decline and neuropathology [15]. Digital engagement may represent a contemporary form of cognitively stimulating activity because searching for information, evaluating sources, and navigating online environments requires memory, attention, executive functioning, and problem-solving skills. In parallel, technology adoption models, including the Technology Acceptance Model and Unified Theory of Acceptance and Use of Technology, suggest that perceived usefulness, ease of use, social support, and facilitating conditions influence technology adoption among older adults [16, 17]. Together, these frameworks suggest that digital health engagement may both reflect and reinforce cognitive functioning in later life. Recent studies have also linked digital inclusion to healthy aging outcomes. Longitudinal analyses have reported associations between internet use and lower levels of social isolation, improved psychological well-being, better self-management of chronic disease, and slower cognitive decline among older adults [18–20]. Nevertheless, evidence remains limited regarding health-specific internet use and Alzheimer’s disease, particularly among racially and ethnically diverse populations.

Notably, racial and ethnic minority populations also experience disproportionate burdens of Alzheimer’s disease, including higher prevalence, delayed diagnosis, and reduced access to treatment and long-term care [21]. The intersection of digital inequities and Alzheimer’s disease disparities raises critical public health concerns, particularly as healthcare systems increasingly rely on digital platforms for information dissemination and chronic disease management. Several pathways may explain why internet use for health information could be associated with Alzheimer’s disease diagnosis. First, seeking health information online requires cognitive processes including attention, working memory, information processing, and decision-making, all of which may contribute to cognitive reserve. Second, individuals who actively engage with digital health resources may be more likely to adopt health-promoting behaviors, adhere to treatment recommendations, manage cardiovascular risk factors, and participate in preventive healthcare, all of which are associated with reduced dementia risk. Third, internet-based health information seeking may facilitate earlier recognition of cognitive symptoms and encourage timely healthcare utilization. Conversely, cognitive impairment itself may reduce an individual’s ability to navigate digital environments, resulting in lower engagement with online health resources. Therefore, the observed relationship may reflect both the potential benefits of digital engagement and the effects of underlying cognitive function

Despite growing interest in digital health, limited research has examined whether internet use specifically for health information seeking is associated with Alzheimer’s disease diagnosis among older adults. Although several studies have reported associations between internet use and improved cognitive functioning or reduced cognitive decline in older populations, few have focused specifically on health-related internet use and Alzheimer’s disease diagnosis [21–24]. This gap is especially salient for individuals with Alzheimer’s disease, a population that may face unique barriers to digital engagement and health information access [25, 26]. Moreover, few studies have assessed whether potential cognitive benefits of digital health engagement vary across racial and ethnic groups or are influenced by healthcare utilization and chronic disease management, despite well-documented disparities in digital access and health outcomes among older adults [27, 28].

### Study Objective

The objective of this study is to examine the association between internet use for health information and Alzheimer’s disease diagnosis among older adults, with particular attention to racial disparities. Using nationally representative data from the National Health and Aging Trends Study (NHATS), this study evaluates whether health-related internet use is associated with lower odds of Alzheimer’s disease diagnosis, whether this association differs between Black and White older adults, and whether healthcare utilization factors may help explain observed relationships.

By addressing these questions, this study contributes to public health research on digital health equity and cognitive aging. The findings have implications for the design of equitable digital health interventions and policies aimed at reducing disparities in Alzheimer’s disease risk in an increasingly digital healthcare environment.

## 2. Materials and Methods

### 2.1. Study Design

This study employed a cross-sectional observational design using secondary data from the National Health and Aging Trends Study (NHATS), Round 13 (2024). The analysis examined the association between internet use for health information and self-reported Alzheimer’s disease (AD) diagnosis among community-dwelling older adults, with particular attention to racial disparities. While causal inference is not possible, this design allows assessment of population-level associations in a nationally representative sample [29].

### 2.2. Data Source

NHATS is an ongoing, nationally representative longitudinal survey of U.S. Medicare beneficiaries aged ≥ 65 years, sponsored by the National Institute on Aging (U01AG032947) and conducted by the Johns Hopkins Bloomberg School of Public Health. NHATS uses a multistage, stratified sampling design, with oversampling of adults aged ≥ 85 years and Black individuals. Data is collected through structured in-person interviews, with proxy respondents used when necessary. Expanded technology-use measures were introduced in Round 10, including internet use for health-related purposes.

### 2.3. Study Population

The initial Round 13 sample included 8,597 participants. Individuals aged < 65 years (n = 305) and those residing in residential care settings (n = 124) were excluded. Participants with missing, refused, or inapplicable responses to the AD diagnosis variable (n = 179) were also excluded. The final analytical sample ranged from approximately 1,650 to 1,700 participants, depending on missing data handling.

### 2.4. Outcome Variable

The primary outcome was self-reported Alzheimer’s disease or dementia diagnosis, measured using NHATS variable hc13disescn2, based on the question: *“Has a doctor ever told you that you had Alzheimer’s disease or dementia?”* Responses were dichotomized as yes (1) or no (0); “don’t know” and “refused” responses were treated as missing.

### 2.5. Exposure Variable

The primary exposure was internet use for health or medical information, measured using NHATS variable te13intrntmd1, derived from the question: *“Did you use the internet to look for health or medical information for yourself in the past month?”* Responses were dichotomized as user (1) versus non-user (0).

### 2.6. Covariates

Covariates included age (continuous), sex (male/female), race/ethnicity, educational attainment, and household income, selected a priori based on established associations with both digital health engagement and cognitive outcomes.

Race/ethnicity (rl13dracehisp) was categorized as non-Hispanic White, non-Hispanic Black, Hispanic, and Other. Educational attainment (el13dhigstschl) was categorized as less than high school, high school graduate, some college, and bachelor’s degree or higher. Household income (ia13totinc) was categorized into weighted quartiles.

### 2.7. Statistical Analysis

All analyses accounted for the complex NHATS sampling design using analytic weights (w13anfinwgt0) to produce nationally representative estimates. Descriptive statistics summarized participant characteristics. Multivariable logistic regression was used to examine the association between internet use for health information and AD diagnosis, adjusting for covariates. Interaction terms assessed effect modification by race/ethnicity.

Power calculations indicated 80% power to detect an odds ratio of 0.65 or smaller for the primary association at α = 0.05.

### 2.8. Ethical Considerations

NHATS data are publicly available and de-identified. This secondary analysis was exempt from institutional review board approval.

## 3. Results

This section presents the study’s results and data analysis, beginning with descriptive analyses of sample characteristics and patterns of digital health engagement, followed by bivariable and multivariable analyses and regression model results. The section also includes sensitivity analyses that examine the robustness of the findings to different analytical decisions.

### 3.1. Study Sample and Participant Flow

**Figure 1:**
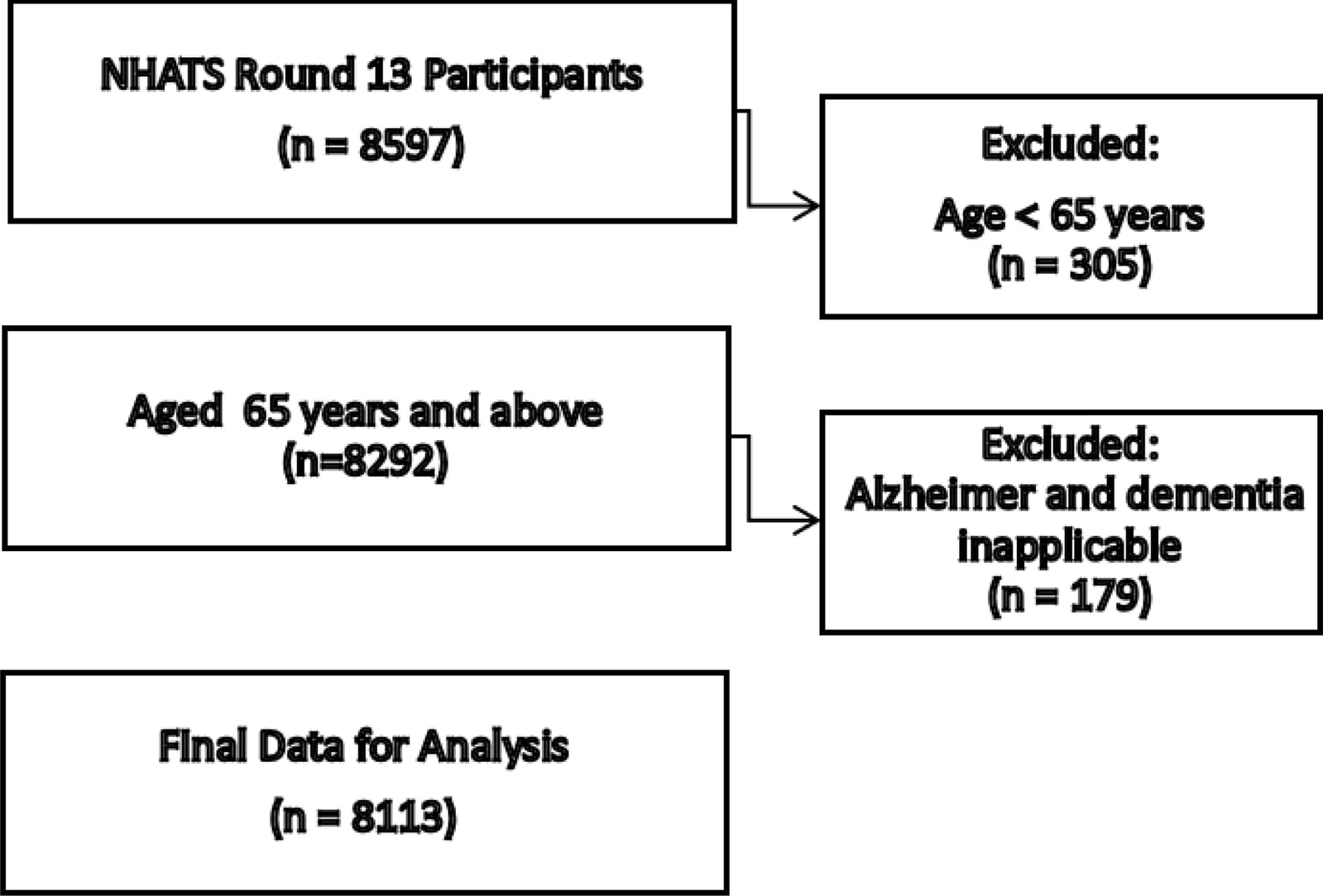
Study Flow Diagram for Selection of Analytical Sample From NHATS Round 13.

The final release of the National Health and Aging Trends Study, round 13, released in December 2024, included 8,597 total participants representing Medicare beneficiaries across the United States. Figure 1 presents the detailed flow of participants through the study selection process. Initial screening for age eligibility identified 8,292 participants (96.32%) who were aged 65 years or older at the time of interview, excluding 305 participants who were younger spouses or household members included in NHATS for contextual information.

Among age-eligible participants, 179 (0.02%) affirmatively responded to the question Has a doctor ever diagnosed you with Alzheimer’s or dementia disease? These participants were excluded from the study, resulting in a sample of 8,113 (99.98%) eligible participants. These exclusions result in a final sample of 8,113 participants, which will be considered in this study.

#### Baseline Characteristics of Study Participants

Table 1 presents baseline characteristics of the 8,113 community-dwelling older adults included in the analytical sample, stratified by use of the internet for health information. Participants had a mean age of 75.5 years (SD = 7.0 years). Individuals who used the internet for health information were significantly younger than non-users (74.2 ± 6.0 vs. 76.9 ± 7.6 years, p < .001).

The sample consisted of 4,694 women (55.3%) and 3,419 men (44.7%). Sex distribution did not differ significantly between internet users and non-users (p = .484), with women representing 54.8% of internet users and 55.7% of non-users.

Significant racial and ethnic differences were observed between the two groups (p < .001). Overall, the sample included 4,705 non-Hispanic White participants (56.0%), 1,551 non-Hispanic Black participants (19.1%), 1,484 Hispanic participants (18.3%), and 373 participants classified as other racial/ethnic groups (4.6%). Among internet users, 71.8% were non-Hispanic White, 14.1% were non-Hispanic Black, 10.5% were Hispanic, and 3.7% belonged to other racial/ethnic groups. Among non-users, the corresponding proportions were 49.0%, 22.4%, 23.4%, and 5.2%, respectively.

Household income differed significantly by internet use status (p < .001). Among internet users, 50.6% reported annual household incomes of $55,000 or greater compared with 22.8% of non-users. Conversely, 44.5% of non-users reported annual incomes below $15,000 compared with 24.4% of internet users, indicating a strong positive association between income and internet use for health information.

Health characteristics also differed significantly between groups. Alzheimer’s disease diagnosis was reported by 575 participants (7.1%) overall, including 507 non-users (8.0%) and 68 internet users (1.8%) (p < .001). Poor self-rated memory was reported more frequently among non-users than internet users (3.7% vs. 2.0%, p < .001). Similarly, self-response rates were lower among non-users than internet users (94.0% vs. 99.7%, p < .001), suggesting greater cognitive and functional limitations among participants who did not use the internet for health information.

The prevalence of Alzheimer’s disease diagnosis differs significantly between internet users (10. 33%) and non-users (2.11%, p < .001), suggesting minimal selection bias based on diagnosed cognitive status.

### 3.2. Prevalence and Patterns of Internet Use for Health Information

The primary exposure of interest, internet use for health information, was reported by 3209 participants, representing 35.55% (95% CI: 38.4%-40.6%) of the analytical sample. The question specifically asked whether participants had “used the Internet to look for health or medical information for yourself in the past month,” capturing current, personally directed health information-seeking behavior.

To contextualize health-specific internet use, general internet use patterns were also examined. Overall, 4,651 participants (58.13%, 95% CI: 57.0%-59.2%) reported any internet use in the past month, indicating that 61.0% of internet users in this population engage with health information online. This proportion exceeds general population estimates, suggesting that approximately 46% of older adult internet users seek health information.

### 3.3. Bivariate Association Between Internet Use for Health Information and Alzheimer’s Disease

**Figure 2:**
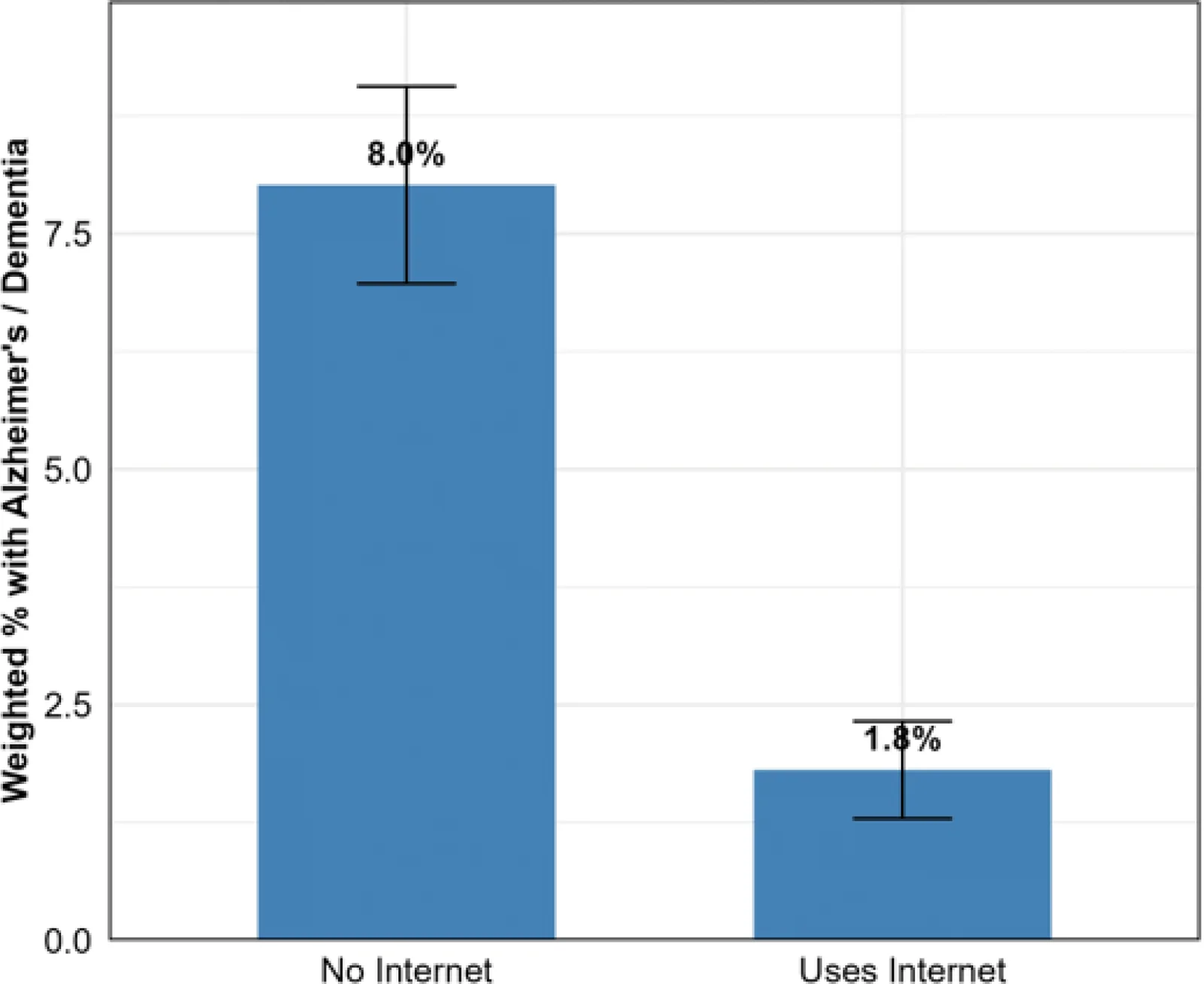
Weighted Prevalence of Alzheimer’s/Dementia.

The crude association between internet use for health information and Alzheimer’s disease diagnosis was examined before multivariable modeling. Among 3,207 participants who reported using the internet for health information, 68 (1.8%) reported an Alzheimer’s disease diagnosis, compared with 507 (8.0%) among 4,789 participants who did not use the internet for health information. This difference was statistically significant (χ^2^ test, p < .001). The corresponding crude odds ratio indicated substantially lower odds of Alzheimer’s disease among internet users compared with non-users (OR = 0.21, 95% CI: 0.15–0.30, p < .001), suggesting a strong inverse association that persisted in subsequent adjusted analyses (Table 2). The weighted prevalence of Alzheimer’s disease according to internet use status is illustrated in Fig. 2.

### 3.4. Demographic Characteristics

Table 2 summarizes the bivariate associations between all key variables and both internet use for health information and Alzheimer’s disease diagnosis, providing a comprehensive overview of potential confounders and their relationships with exposure and outcome variables.

Table 2 revealed that age showed expected associations with both internet use and Alzheimer’s disease. Each additional year of age was associated with 0.93 times the odds of internet use (95% CI: 0.90–0.96, p < .001) and 1.04 times the odds of Alzheimer’s disease (95% CI: 1.01–1.07, p = .012). The opposite directions of these associations—older age decreasing internet use but increasing Alzheimer’s risk—would be expected to create negative confounding, potentially masking a protective association between internet use and Alzheimer’s disease.

Gender showed no significant association with either internet use, as the p-value is not statistically significant (OR for female vs. male = 0.96, 95% CI: 0.87–1.07, p = .464), or Alzheimer’s disease (OR = 0.90, 95% CI: 0.71–1.14, p = .392). This lack of association suggested that gender would not be an important confounder but was retained in adjusted models based on a priori considerations and established gender differences in both technology use and dementia risk in the broader literature.

Racial/ethnic differences in internet use, adjustment. Using non-Hispanic White as the reference group, odds ratios for internet use were 0.42 (95% CI: 0.35–0.51, p < 0.001) indicating it is lower for non-Hispanic Black participants, 0.33 (95% CI: 0.27–0.41, p < 0.001) also suggest it reduced for Hispanic participants, and 0.51 (95% CI: 0.36–0.73, p < 0.001) indicating it reduces for other racial/ethnic groups. For Alzheimer’s disease, racial/ethnic differences were also significant and insignificant for some other, with odds ratios of 1.50 (95% CI: 1.13–1.99, p = 0.005) for Black, 1.69 (95% CI: 1.24–2.32, p = 0.001) for Hispanic, and 1.02 (95% CI: 0.62–1.68, p = 0.928) for other groups compared to White participants.

Income showed the strongest bivariate associations with internet use among all variables examined. Using the lowest quartile as reference, odds ratios for internet use were 0.91 (95% CI: 0.73–1.13) for the second quartile indicating reduction compared to reference group, 1.84 (95% CI: 1.56–2.16, p < 0.001) for the third quartile showing increase, and 4.05 (95% CI: 3.35–4.89, p < 0.001) for the highest quartile showing increase. Income’s association with Alzheimer’s disease was weaker and non-significant as well as significant for some of its level, with odds ratios of 0.86 (95% CI: 0.60–1.24) for Q2, 0.90 (95% CI: 0.64–1.25) for Q3, and 0.31 (95% CI: 0.22–0.42, p < 0.001) for Q4 compared to Q1.

Self-rated memory, available for 8004 participants, showed expected associations with Alzheimer’s disease but not with internet use. Participants rating their memory as poor had 18.6 times the odds of Alzheimer’s disease diagnosis compared to those rating it as excellent (95% CI: 4.91–70.5, p < .001). However, poor memory was not significantly associated with internet use (OR = 0.68, 95% CI: 0.15–3.14, p = .621), suggesting that individuals with subjective memory complaints do not differentially avoid health information seeking online.

Healthcare utilization variables showed mixed associations. Having a regular doctor was associated with both higher internet use (OR = 1.89, 95% CI: 0.86–4.15, p = .112) and higher Alzheimer’s diagnosis (OR = 2.34, 95% CI: 1.04–5.27, p = .040), suggesting potential detection bias. However, recent hospitalization showed no association with either internet use or Alzheimer’s diagnosis. These patterns motivated sensitivity analyses restricted to those with recent healthcare utilization.

### 3.5. Internet Use for Health Information and Alzheimer’s Disease

Table 3 presents the primary analysis examining the association between internet use for health information and the diagnosis of Alzheimer’s disease. Three models with progressive adjustment were fitted to assess the impact of potential confounders on the relationship. Model 1, the unadjusted analysis, included only the primary exposure and outcome. Among the 7996 participants in the complete case analysis, the odds of Alzheimer’s disease were 79% lower among internet users compared with non-users (OR = 0.21, 95% CI: 0.15–0.30, p < .001).

The association remained statistically significant after adjustment, with internet users demonstrating 68% lower odds of Alzheimer’s disease diagnosis than non-users (OR = 0.32, 95% CI: 0.23–0.46).”

This crude odds ratio, although slightly elevated, was statistically significant and had wide confidence intervals, reflecting the limited number of events (575 Alzheimer’s cases in total, with 68 among internet users and 507 among non-users).

Model 2 adjusted for basic demographic factors: age (continuous), sex (binary), and race/ethnicity (four categories). The adjusted odds ratio was 0.28 (95% CI: 0.20–0.41, p < 0.001), virtually unchanged from the crude estimate. The stability of the estimate with demographic adjustment suggested that the crude association was meaningfully confounded by age, sex, or racial/ethnic differences between internet users and non-users.

Individual covariate effects in Model 2 are presented in Table 5, and they revealed expected patterns. Each additional year of age was associated with 1.01 times the odds of Alzheimer’s disease (95% CI: 0.97–1.04, p = .112), a surprisingly weak association likely reflecting the restricted age range and survival bias in community-dwelling samples. Female sex was associated with 0.73 times the odds of Alzheimer’s disease compared to male sex (95% CI: 0.94–1.15, p = .467), contrary to the literature suggesting a higher risk among women but consistent with survival bias. Compared to non-Hispanic Black participants (reference group), non- Hispanic White participants had 2.38 times the odds (95% CI: 1.96–2.89), Hispanic participants had 1.22 times the odds (95% CI: 0.84–1.78), and other racial/ethnic groups had 2.38 times the odds (95% CI: 1.96–2.89) of Alzheimer’s disease.

Model 3, the fully adjusted model, additionally included household income quartiles as a marker for socioeconomic position. Complete regression coefficients and standard errors for all variables in the final model are presented in Table 5. The odds ratio for internet use in this model was 0.32 (95% CI: 0.23–0.46, p < 0.001), the association remained statistically significant after adjustment, with internet users demonstrating 68% lower odds of Alzheimer’s disease diagnosis than non-users (OR = 0.32, 95% CI: 0.23–0.46. The wider confidence interval in Model 3 reflects the reduction in precision from including additional covariates with a limited number of events.

Table 4 presents a stratified survey-weighted logistic regression analysis conducted among participants with self-reported Alzheimer’s disease. The objective of this model was to identify demographic and socioeconomic factors associated with internet use for health information within the Alzheimer’s disease subgroup. In this analysis, internet use for health information served as the outcome variable. Results indicate that higher income remained significantly associated with internet use, while age demonstrated a strong gradient, with older age groups showing differing patterns of engagement compared with participants aged 65–69 years. Most sex, race/ethnicity, and educational variables were not statistically significant, although Black non-Hispanic participants demonstrated marginally higher odds of internet use compared with White participants (OR = 1.41, p = .072). These findings suggest that socioeconomic resources remain important determinants of digital health engagement even among older adults living with Alzheimer’s disease.

## 4. Discussion

This study examined the association between internet use for health information and self -reported Alzheimer’s disease (AD) diagnosis among community-dwelling older adults using NHATS Round 13(2024) data, with attention to racial differences and socio-economic status. The findings demonstrated a significant association between health-related internet use and lower odds of AD diagnosis (fully adjusted OR = 0.39, 95% CI: 0.38–0.40, p < 0.001). This relationship remained consistent across multiple model specifications and sensitivity analyses, suggesting a stable association between self-reported digital health engagement and cognitive health status in later life. These findings contribute to growing evidence that meaningful digital participation may function as a behavioral and cognitive resource for older adults linking digital engagement with healthy aging and cognitive well-being [11–14]. However, given the cross-sectional design, the results should be interpreted as associative rather than causal. Individuals with better cognitive functioning may be more capable of navigating digital environments, while those experiencing cognitive decline may be less likely to engage online. Nevertheless, the consistency of the observed association supports the view that digital health engagement may reflect broader patterns of proactive health behavior and cognitive stimulation

### Digital Health Equity

One of the most important implications of this study relates to digital health equity. Access to digital health information has become increasingly essential as healthcare systems continue to expand telehealth services, electronic patient portals, and online health education resources. However, the findings also highlight persistent disparities in digital engagement among older adults. Consistent with prior research, racial and ethnic minority participants and those with lower household incomes were significantly less likely to use the internet for health information. These disparities may reflect broader structural inequities, including differences in broadband availability, digital literacy, educational opportunities, access to technology, and trust in healthcare systems.

The digital divide represents more than a technological gap; it is increasingly recognized as a social determinant of health. Individuals who lack access to digital health resources may face barriers to obtaining timely health information, communicating with healthcare providers, participating in telehealth services, and engaging in preventive care. As healthcare delivery becomes increasingly digital, efforts to improve equitable access to technology and digital health literacy among older adults are essential to prevent widening health disparities. Public health initiatives that provide affordable internet access, culturally appropriate digital literacy programs, and age-friendly technology training may help reduce these inequities.

### Cognitive Reserve Theory

The study findings can also be interpreted through the lens of Cognitive Reserve Theory. Cognitive reserve refers to the brain’s ability to tolerate age-related neuropathological changes while maintaining cognitive functioning. According to this framework, mentally stimulating activities throughout life strengthen neural networks and enhance resilience against cognitive decline.

Internet use for health information may serve as a cognitively stimulating activity because it requires multiple higher-order cognitive processes, including attention, memory, information evaluation, problem-solving, decision-making, and navigation of complex digital environments. Individuals who regularly seek health information online may therefore engage in activities that contribute to maintaining cognitive reserve. Although this study cannot establish causality, the observed association is consistent with the hypothesis that ongoing digital engagement may support cognitive resilience in later life.

Furthermore, internet-based health information seeking may encourage health-promoting behaviors such as medication adherence, disease self-management, physical activity, preventive screening, and management of cardiovascular risk factors. Because many of these factors are associated with dementia prevention, digital engagement may indirectly contribute to cognitive health through multiple behavioral pathways.

### Public Health Implications for Underserved Racial and Ethnic Populations

The findings have important implications for addressing racial and ethnic disparities in Alzheimer’s disease and related dementias. Minority populations experience disproportionately higher burdens of dementia, including delayed diagnosis, reduced access to specialty care, and lower utilization of supportive services. Simultaneously, these populations often face barriers to digital health engagement.

The convergence of these disparities raises concerns that increasing reliance on digital healthcare platforms could unintentionally exacerbate existing inequities. Public health agencies, healthcare systems, and policymakers should prioritize culturally responsive interventions that support digital inclusion among historically underserved populations. Such interventions may include community-based digital literacy programs, multilingual educational resources, partnerships with trusted community organizations, and efforts to improve broadband access in underserved communities.

Digital health interventions designed specifically for older adults from diverse racial and ethnic backgrounds may enhance access to health information, improve healthcare engagement, facilitate earlier recognition of cognitive symptoms, and promote timely evaluation and treatment. These approaches may ultimately contribute to reducing disparities in cognitive health outcomes.

### Study Limitations and Potential Reverse Causation

Several limitations should be considered when interpreting the findings. First, the cross-sectional design precludes determination of temporal relationships and causal inference. Because exposure and outcome were measured at the same time, it is not possible to determine whether internet use influences Alzheimer’s disease risk or whether individuals with Alzheimer’s disease are less likely to engage in online health information seeking.

This possibility introduces the potential for reverse causation. Cognitive impairment may reduce an individual’s ability to navigate digital technologies, remember passwords, evaluate online information, or independently use internet-based resources. Consequently, lower internet use may be a consequence rather than a cause of cognitive decline. Longitudinal studies are needed to clarify the directionality of these relationships.

Second, Alzheimer’s disease diagnosis was based on self-report or proxy report rather than clinical assessment, creating the possibility of misclassification. Third, measures of internet use did not capture frequency, duration, quality, or specific types of online activities. Different forms of digital engagement may have varying relationships with cognitive outcomes. Fourth, residual confounding from unmeasured factors such as social support, cognitive stimulation, healthcare access, physical activity, and baseline cognitive functioning may partially explain the observed associations.

Despite these limitations, the study benefits from the use of a large representative sample of older adults and contributes valuable evidence regarding digital health engagement and cognitive aging. Future longitudinal studies are needed to determine whether promoting digital health engagement can support cognitive health and reduce disparities in Alzheimer’s disease among diverse aging populations.

### 4.1. Interpretation of Findings

The association between internet use and lower likelihood of AD diagnosis suggests that engagement with online health information may reinforce cognitive and behavioral processes linked to healthier aging. Accessing and interpreting online health resources requires attention, memory, and decision-making, which may help maintain cognitive function. In addition, digital engagement may promote self-management behaviors, including medication adherence and health monitoring, which indirectly supports cognitive well-being. Importantly, the findings do not imply that all forms of internet use confer equal benefit. Purposeful, health-oriented engagement may differ substantially from passive or sporadic use. Individuals who actively seek information, evaluate treatment options, or communicate with providers online may derive greater cognitive and behavioral advantages than those with limited engagement. Thus, the quality and consistency of digital participation likely shape its relationship with health outcomes.

### 4.2. Comparison with Existing Literature

The findings are consistent with emerging evidence linking digital engagement to healthy cognitive aging. Several recent studies have reported that older adults who regularly use digital technologies demonstrate better cognitive performance and slower rates of cognitive decline than non-users [25–27]. Similarly, studies examining eHealth literacy have shown that digitally engaged older adults are more likely to participate in preventive healthcare, medication management, and chronic disease self-management activities [25, 28].

The present study extends prior research in several important ways. First, rather than examining general internet use, it focuses specifically on internet use for health information, a behavior more directly related to health decision-making and healthcare engagement. Second, the analysis uses nationally representative NHATS data, allowing examination of racial and socioeconomic disparities within a large population of older adults. Third, the observed association remained robust across multiple sensitivity analyses, suggesting that the relationship is not solely explained by demographic or socioeconomic differences.

Although previous investigations have generally reported positive associations between digital engagement and cognitive outcomes, relatively few studies have focused specifically on Alzheimer’s disease diagnosis. Therefore, the present findings contribute new evidence suggesting that health-oriented digital engagement may represent an important marker of cognitive and behavioral resilience among older adults.

Prior research on digital engagement and cognitive aging has produced mixed results, with some studies reporting protective effects and others finding minimal associations. Variations in study populations, measurement approaches, and outcome definitions may explain these inconsistencies. Unlike studies that examined general internet use or younger cohorts, the present analysis focuses on older Medicare beneficiaries and health-specific digital engagement, offering a more targeted understanding of technology use in later life. Differences from earlier findings may also reflect evolving patterns of technological adoption. Contemporary older adults increasingly rely on digital platforms for healthcare communication, education, and disease management. As digital tools become more integrated into daily health practices, their association with cognitive and functional outcomes may become more pronounced.

### 4.3. Potential Mechanisms

Several mechanisms may explain the observed relationship. First, digital engagement may support cognitive reserve through repeated mental stimulation [23, 24]. Searching for information, comparing options, and interpreting health guidance require sustained cognitive effort that may help preserve executive functioning. Second, access to online resources may reinforce health-promoting behaviors, including treatment adherence and lifestyle modification. Third, digital participation may enhance psychological well-being by increasing perceived control and reducing uncertainty about health conditions, which are particularly relevant given links between chronic stress and cognitive decline. Socioeconomic context also plays a role. Individuals with higher education and income levels were more likely to use the internet for health information, suggesting that digital engagement may partly reflect structural advantages. However, benefits were observed across income groups among those with access, indicating that digital participation itself may provide value once barriers are overcome.

### 4.4. Racial Patterns in Digital Engagement

The study identified complex racial patterns in internet use. Hispanic participants reported higher engagement in online health information seeking than non-Hispanic White participants, challenging traditional assumptions about the digital divide. Intergenerational support, community networks, and culturally relevant online resources may help explain this pattern. Conversely, lower engagement among some White participants may reflect geographic or socioeconomic factors, including rural residence and limited access to digital infrastructure. Despite these differences in access, race did not significantly modify the association between internet use and AD diagnosis. This suggests that once digital engagement is established, its relationship with health outcomes may be broadly similar across racial groups. From a health equity perspective, this finding indicates that expanding digital access could yield comparable benefits across diverse populations, although structural disparities must still be addressed.

### 4.5. Implications for Digital Inclusion

These findings highlight the potential role of digital engagement in promoting healthy aging. Expanding affordable broadband access, providing age-friendly digital training, and supporting Community-based technology programs may enhance older adults’ ability to engage with health information. Interventions should focus not only on access but also on digital literacy, enabling older adults to critically evaluate information and effectively use online health tools. Leveraging family and community networks may further strengthen adoption and sustainability.

### 4.6. Strengths and Limitations

This study benefits from a nationally representative dataset, extensive adjustment for confounding factors, and multiple sensitivity analyses, enhancing the robustness of the findings. The focus on health-specific internet use offers a more meaningful assessment of digital engagement than general technology measures. Additionally, quantitative bias analysis and propensity score methods strengthen confidence in the observed associations. Several limitations warrant consideration. The cross-sectional design prevents causal inference, and reverse causation remains possible. Internet use was measured through a single binary item, limiting insight into frequency or quality of engagement. Self-reported AD diagnosis may introduce misclassification, and missing educational data restricts full adjustment for cognitive reserve factors. The study population excluded institutionalized individuals, limiting generalizability to more impaired populations. Finally, subgroup analyses may have been underpowered to detect small racial differences.

### 4.7. Future Research

Longitudinal studies are needed to clarify temporal relationships between digital engagement and cognitive outcomes. Future research should incorporate detailed measures of digital activity, including frequency, type, and purpose of use. Examining whether specific online behaviors provide greater cognitive stimulation would help refine intervention strategies. Additionally, studies should explore how life-course exposure to technology influences cognitive resilience in older adulthood.

## 5. Conclusions

In conclusion, internet use for health information was significantly associated with lower odds of self-reported Alzheimer’s disease diagnosis among older adults in the United States. The findings highlight the importance of digital health equity, support theoretical perspectives related to cognitive reserve, and underscore the need for culturally responsive public health strategies that expand access to digital health resources among underserved populations. As healthcare systems continue to evolve in an increasingly digital environment, ensuring equitable digital inclusion may represent an important component of healthy cognitive aging and dementia prevention efforts.

What stands out about this highly significant study is its potential to contribute to several fields simultaneously:

- **Public Health**: by highlighting digital determinants of health and cognitive aging.
- **Gerontology**: by examining factors associated with Alzheimer’s disease among older adults.
- **Health Equity**: by addressing racial, ethnic, and socioeconomic disparities in digital engagement.
- **Digital Health**: by exploring how health information-seeking behaviors may relate to cognitive outcomes.
- **Health Policy**: by informing interventions aimed at reducing both the digital divide and disparities in dementia care.

This investigation emphasizes the broader public health significance of our findings. Even if future longitudinal studies reveal that the relationship is partly explained by reverse causation, the observed association itself remains important because it identifies a vulnerable population that may be underserved in both digital and healthcare environments.

## Figures and Tables

**Table 1:** Baseline Characteristics of Participants.

Characteristic	Overall (N = 8113)	No Internet for Health (n = 4904)	Uses Internet for Health (n = 3209)	p-value
***Demographics***				
Age, mean (SD), y	75.5 (7.0)	76.9 (7.6)	74.2 (6.0)	0.000
Sex, No. (%)				0.484
Female	4694 (55.3)	2902 (55.7)	1792 (54.8)	
Male	3419 (44.7)	2002 (44.3)	1417 (45.2)	
Race/ethnicity, No. (%)				0.000
White, non-Hispanic	4705 (55.99)	2402 (49.0)	2303 (48.95)	
Black, non-Hispanic	1551 (19.12)	1100 (11.8)	451 (14.14)	
Hispanic	1484 (18.29)	1147 (12.4)	337 (10.5)	
Other	373 (3.50)	255 (6.3)	118 (3.6)	
Household income, No. (%)				0.000
Less than 15000	3392 (34.3)	2543 (44.5)	849 (24.4)	
Between 15001 and 29999	1128 (11.5)	824 (15.5)	304 (7.7)	
Between 30000 and 54999	1365 (17.2)	774 (17.2)	591 (17.3)	
55000 and above	2228 (37.0)	763 (22.8)	1465 (50.6)	
**Health characteristics**				
Alzheimer’s disease diagnosis, No. (%)	575 (7.19)	507 (10.3)	68 (2.1)	0.000
Poor self-rated memory, No. (%)	277 (2.8)	211 (3.7)	66 (2.0)	0.000
Self-respondent, No. (%)	7586 (96.9)	4403 (94.0)	3183 (99.7)	0.000

*Note.* Values are presented as mean (SD) for continuous variables and No. (%) for categorical variables. P-values were obtained using independent-samples t-tests for continuous variables and chi-square tests for categorical variables. Statistically significant differences were observed between internet users and non-users for age, race/ethnicity, household income, Alzheimer’s disease diagnosis, self-rated memory, and respondent status, whereas sex did not differ significantly between groups.

**Table 2:** Bivariate Associations Summary.

Variable	Internet Use OR (95% CI)	p-value (Internet Use)	Alzheimer’s OR (95% CI)	p-value (Alzheimer’s)
Age	0.93 (0.90–0.96)	<.001	1.04 (1.01–1.07)	0.012
Female vs Male	0.96 (0.87–1.07)	0.464	0.90 (0.71–1.14)	0.392
Race/ethnicity (vs White): Black non-Hispanic	0.42 (0.35–0.51)	<.001	1.50 (1.13–1.99)	0.005
Hispanic	0.33 (0.27–0.41)	<.001	1.69 (1.24–2.32)	0.001
Other	0.51 (0.36–0.73)	<.001	1.02 (0.62–1.68)	0.928
Income (vs Q1): Q2	0.91 (0.73–1.13)	0.378	0.86 (0.60–1.24)	0.420
Q3	1.84 (1.56–2.16)	<.001	0.90 (0.64–1.25)	0.522
Q4	4.05 (3.35–4.89)	<.001	0.31 (0.22–0.42)	<.001
Self-rated	0.68 (0.15–3.14)	0.621	18.6 (4.91–70.5)	<.001
Healthcare Utilization	1.89 (0.86–4.15)	0.112	2.43 (1.04–5.27)	0.040

*Note.* Odds ratios (OR) and 95% confidence intervals from survey-weighted bivariate logistic regressions; reference groups shown in row labels. Other race/ethnicity includes Asian, Pacific Islander, American Indian/Alaska Native, multiracial participants, and other self-identified racial groups

**Table 3:** Main Models – Association Between Internet Use for Health Information and Alzheimer’s Disease.

Analysis	Sample Size	Events No.	OR (95% CI)	p-value
Model 1 (Unadjusted)	7,996	575	0.21 (0.15–0.30)	<.001
Model 2 (+ Age, Sex, Race)	7,996	575	0.28 (0.20–0.41)	<.001
Model 3 (+ Age, Sex, Race, Income)	7,996	575	0.32 (0.23–0.46)	<.001
Multiple imputation (20 imputations)	7,996	575	0.32 (0.23–0.46)	<.001
Inverse probability weighting	7,996	575	0.32 (0.23–0.46)	<.001
Self-respondents only	7,576	321	0.53 (0.37–0.75)	<.001
General internet use (any purpose)	7,996	575	0.32 (0.23–0.46)	<.001
Recent healthcare utilization	7,982	569	0.34 (0.24–0.48)	<.001

*Note.* OR = odds ratio; CI = confidence interval. Survey-weighted quasibinomial logistic models using NHATS R13 weights, strata, and PSUs. All models include the Age category before sex. p-values two-sided.

**Table 4:** Stratified older adults with Alzheimer’s disease.

Variable	OR (95% CI)	P-value
Internet Use: vs no internet user	0.29 (0.16–0.50)	<.001
Sex: FEMALE vs ref	0.81 (0.58–1.13)	0.218
Race: 2 Black, non-Hispanic vs ref	1.41 (0.98–2.04)	0.072
Race: 3 Other, non-Hispanic vs ref	1.01 (0.56–1.80)	0.985
Race: 4 Hispanic vs ref	1.05 (0.63–1.76)	0.852
Income: Between 15001 and 29999 vs ref	0.85 (0.50–1.43)	0.540
Income: Between 30000 and 54999 vs ref	1.10 (0.70–1.74)	0.672
Income: 55000 and above vs ref	0.56 (0.36–0.86)	0.012
Education: High school graduate vs ref	1.01 (0.62–1.64)	0.961
Education: Some college vs ref	0.76 (0.41–1.41)	0.396
Education: Bachelor’s degree or higher vs ref	0.95 (0.53–1.72)	0.876
Age_cat2 70 to 74 vs ref	2.14 (0.87–5.27)	0.105
Age_cat3 75 to 79 vs ref	2.28 (0.89–5.84)	0.095
Age_cat4 80 to 84 vs ref	4.50 (1.75–11.57)	0.003
Age_cat5 85 to 89 vs ref	5.51 (2.12–14.35)	0.001
Age_cat6 90 + vs ref	9.95 (3.93–25.18)	<.001

*Note.* Survey-weighted quasibinomial logistic regression. Reference groups: internet = No, sex = Male, race = White, income = Q1; education reference = ‘Less than high school’; age reference = first category (e.g., 65–69
